# Supplementary material for: Social synchronization of brain activity increases during eye-contact
Source: Commun Biol. 2022 May 4;5:412. doi: 10.1038/s42003-022-03352-6 (PMC9068716; doi:10.1038/s42003-022-03352-6)
Supplement: Supplementary file 2 — Supplementary Information [file 42003_2022_3352_MOESM2_ESM.pdf]

Supplementary Notes for

Social synchronisation of brain activity increases during eye-contact

Caroline Di Bernardi Luft[1]*, Ioanna Zioga[1,2], Anastasios Giannopoulos[3], Gabriele Di Bona[4], Nicola Binetti[1], Andrea Civilini[4], Vito Latora[4,5,6,7], Isabelle Mareschal[1]

*Corresponding author

Email: c.luft@qmul.ac.uk

This file includes:

Note 1: Tone-reproduction durations and correlations

Note 2: Emergent leadership behavioural analysis

Note 3: Control analysis on gamma power

Note 4: Undirected network analysis

Note 5: Directed network analysis

Note 6: Local to global efficiency analysis

Supplementary References

Supplementary Note 1: Tone-reproduction durations and correlations

We compared participants' gaze durations during tone reproduction. Specifically, we calculated the mean tone-reproduction duration of each participant (i.e. offset minus onset of eye movement during the tone reproduction), separately in each task and in each trial condition. Gaze durations lower than 0.5 secs or higher than 4 secs were excluded from all analyses. Out of a total of 112 participants (56 pairs), 2 participants were excluded as they had less than 5 trials in at least one of the conditions (due to problems on the eye-tracking calibration).

Eye-contact (EC) vs. control (CTL): we investigated whether the tone-reproduction duration of one participant would be influenced by their pair by conducting a 2 (task: EC vs. CTL) x 2 (tone duration: short vs. long) x 2 (pair duration: same vs. different) repeated measures ANOVA. Note that same duration corresponds to the trials when both partners heard either a short or a long tone, whereas different duration corresponds to trials when one partner heard a short tone and the other partner heard a long tone. We observed a significant main effect of tone duration ($F(1,109) = 574.766$, $p < .001$, $\eta^2 = .841$), long tones induced longer gaze durations than short tones. There was also a main effect of task ($F(1,109) = 66.714$, $p < .001$, $\eta^2 = .380$), with longer gaze times in the control compared to the eye-contact task condition. Results also revealed a significant interaction between tone duration and task ($F(1,109) = 12.498$, $p = .001$, $\eta^2 = .103$). Planned contrasts confirmed that participants gazed longer during long compared to short tones in both tasks ($p < .001$), but that they gazed longer in CTL compared to EC in response to short tones ($t(109) = 6.206$, $p < .001$), as well as in response to long tones ($t(109) = 7.512$, $p < .001$). Interestingly, this analysis revealed a significant interaction between pair duration and tone duration ($F(1,109) = 20.665$, $p < .001$, $\eta^2 = .159$). Planned contrasts showed that participants gazed longer in response to long tones compared to short tones in both pair duration conditions ($p < .001$). Importantly, we observed that participants gazed significantly longer in response to short tones when their partner heard a long tone ($t(109) = 4.305$, $p < .001$), and gazed for less time in response to long tones when the other participant heard a short tone ($t(109) = 2.251$, $p = .026$). These findings reveal that the participants' behaviour was dependent on what their partner was doing (Supplementary Figure 1a).

To ensure that participants were interacting during eye-contact, we tested the correlation between their tone-reproduction durations on trials where both partners were presented with long and short tones in each condition (EC vs. CTL) separately. Four pairs who had less than 5 trials were excluded from this analysis (n = 51 pairs). To test whether the correlation between the intervals estimated between the pairs was higher than chance, we compared the correlation coefficients for each pair in each condition (EC and CTL) against the average correlation coefficient of the same data with their trials shuffled randomly for 1000 iterations. The shuffling was applied within each pair respecting whether they were short or long (so the order of P1 and P2 trials were shuffled independently within each category – short and long). We entered the correlation coefficients in a 2 (task: EC vs. CTL) x 2 (data: real vs. shuffled) within-subjects ANOVA. The permutation analysis revealed a significant difference in the correlation between the tone-reproduction durations in both tasks vs. a shuffled distribution with a 1000 iterations ($F(1,50) = 211.280$, $p < .001$, partial $\eta^2 = .809$). There was a significant effect of task with higher correlations during eye-contact compared to the control task ($F(1,50) = 5.452$, $p = .024$, $\eta^2 = .098$). Importantly, there was a significant interaction between task and data ($F(1,50) = 5.627$, $p = .022$, $\eta^2 = .101$), the correlations were higher during eye-contact compared to control ($t(50) = 2.346$, $p = .022$) and compared to the correlations on the shuffled data ($t(50) = 14.675$, $p < .001$). However, the correlation was also significantly

higher during the control task compared to the shuffled data (t(50) = 10.260, p < .001), evidencing that there is also a good level of behavioural synchronization in the control task (it is important to notice that the participants could still see each other and that one of them could see when the other have finished their time estimation). Most shuffled iterations resulted in positive and negative correlations near zero (Supplementary Figure 1b).

Supplementary Figure 1: Tone reproduction durations and correlations

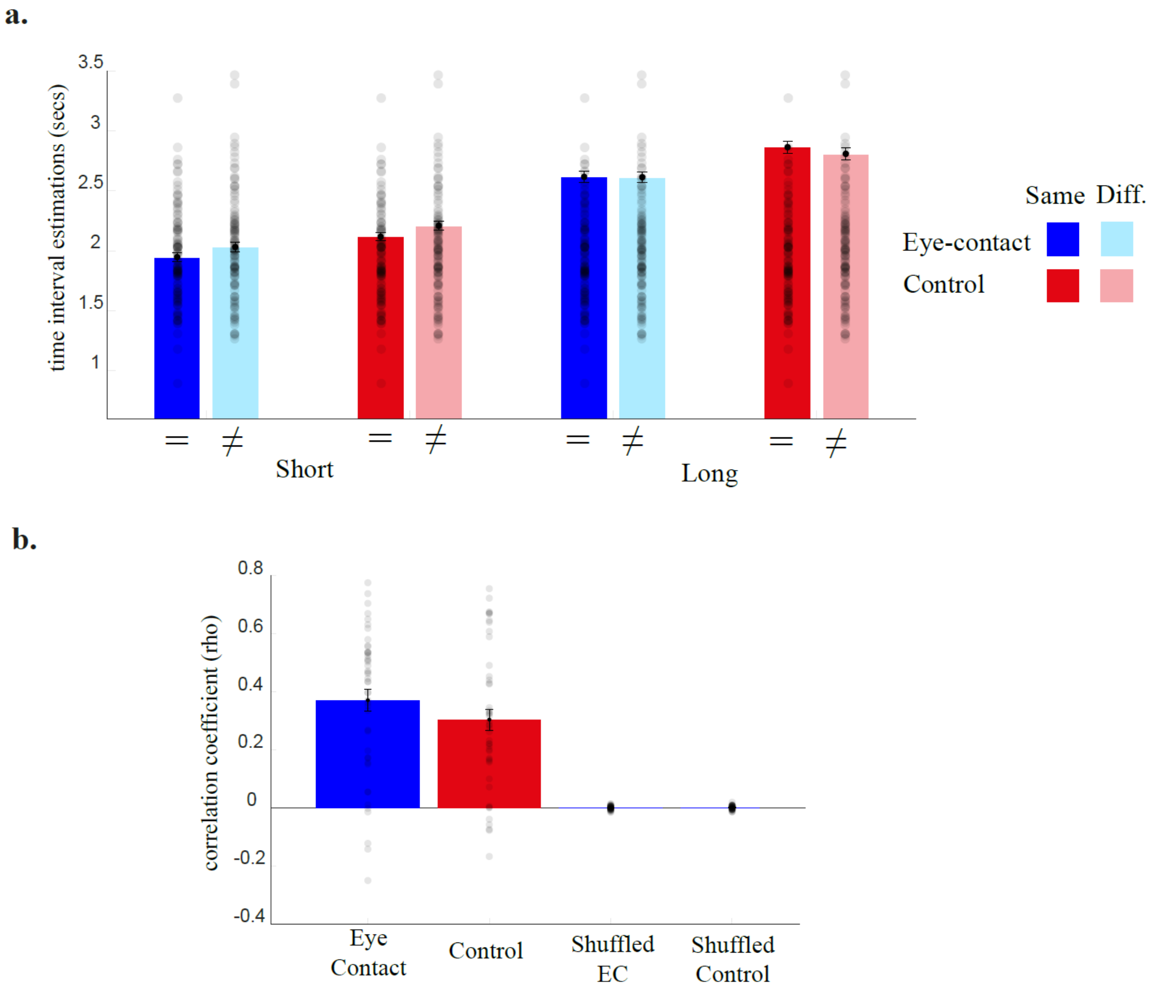

a. Mean tone-reproduction duration (secs) for all pairs, separately for short (left) and long tones (right) during eye contact (EC; blue) vs. control (CTL; red), and for the same duration tones (opaque) compared to different duration tones (transparent); **b.** Average correlations (Spearman's rho) between the tone-reproduction durations of the pairs during eye-contact trials (EC), control trials (CTL) and shuffled trials in the EC and CTL. Shaded dots represent the data for each participant (n = 112) Error bars represent ±1 *SEM*.

Supplementary Note 2: Emergent leadership behavioural analysis

We investigated the nature of the interactions during eye contact by measuring how often one participant gazed down first in the pair. If a participant is being led by the other, they would gaze down after seeing their partner gaze down. If there is no "leading" behaviour, we would observe no such association, with one participant gazing down first approximately 50% of the time. To investigate how leadership roles emerged during the eye-contact task, we identified which participant was the leader and which one was the follower within each pair. Specifically, in each trial, the person who broke eye contact from their partner was considered the leader. We divided the number of trials P1 broke eye contact first compared to the total number of trials. The resulted values spanned from 0 (P2 leads) to 1 (P1 leads). Values around 0.5 meant the absence of a leader (P1 and P2 broke eye-contact in an approximately equal number of trials). As values around both extremes are indicative of strong leadership (e.g., 0.2 signifies the same leadership strength as 0.8; in the first case P2 is the leader, whereas in the second case P1 is the leader), we subtracted all values that were lower than 0.5 from 1. This resulted in values ranging from 0.5 to 1, with higher values indicating stronger leadership pattern. We only examined trials when both participants heard tones with the same duration (both short or both long).

The results showed that for some pairs, one of the participants consistently gazed down first. To ensure that the differences in gazing down did not derive simply from individual differences in time estimation, we compared the leadership strength between participants during eye-contact vs. the same data but with a shuffled order (respecting the participant's position). This way, if a participant consistently gazes down first, shuffling the order of the trials would not make a difference to how many times this participant gazes down first when trying to estimate the same duration (Supplementary Figure S2). We conducted a repeated-measures ANOVA to compare the leadership strength between the eye-contact task, control task and shuffled ordered data (RDM). We observed a significant main effect ($F(2,110) = 4.984$, $p = .008$, partial $\eta^2 = .083$). The leadership strength eye-contact (EC) trials were significantly higher than in shuffled trials, showing that leadership in this task does not solely rely on individual differences in time estimation ($t(55) = 4.081$, $p < .001$). The leadership strength during the control task was marginally higher than the shuffled data ($t(55) = 1.728$, $p = .090$), which makes sense as the participants only had partial access to their partner's eye-movements.

It is important to note that some participants did not present a clear leadership pattern, with values close to chance levels compared to the random distribution (Supplementary Figure S2b). The results from the random distribution show that the chance levels are not 50%, which is probably due to some overlap in their estimations as we shuffled keeping the same participant pair (only the order of the trials was shuffled). Because of this, we split the groups using a median split (median leadership strength = 0.65), which resulted in 27 pairs of participants with what we called a "weak leadership" vs. 29 with a "strong leadership" (>0.65). One pair only presented exactly the same number of trials in which each member gazed down first (50% leadership strength). Because we could not identify a leader and a follower, this pair was excluded from the directed connectivity analysis.

Supplementary Figure 2: Leadership Strength.

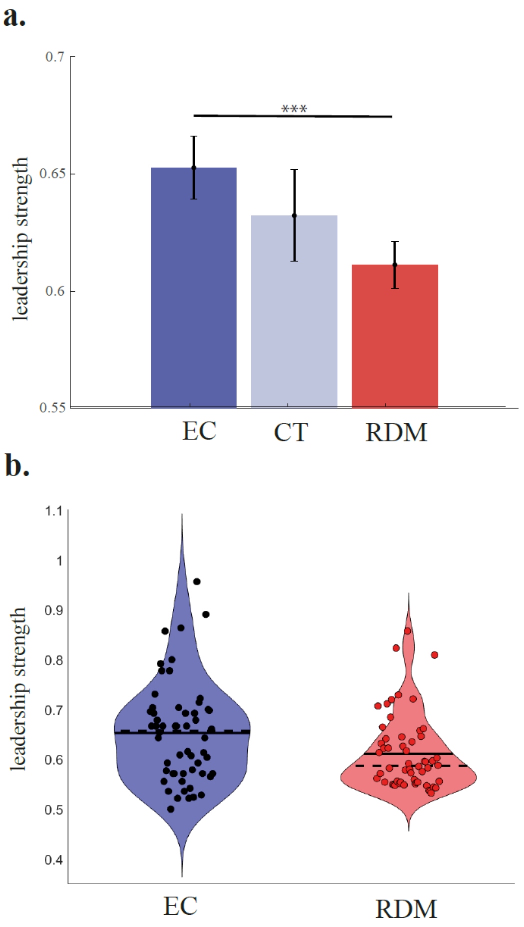

a. Mean leadership strength during eye-contact, control task and on the shuffled data (shuffled order per pair). **b.** Violin plots showing the distribution of the leadership strength during eye-contact (EC) and in the shuffled trials (RDM). The results showed that the chance levels are above 0.5, suggesting some effect of individual differences on time perception. Error bars represent ±1 SEM. *** $p < .001$.

Supplementary Note 3: Control analysis on gamma power

To ensure that our findings were not due to differences in gamma power between the conditions (eye-contact vs. control), we compared gamma power between conditions. We estimated the power spectral density using Welch periodogram. For each participant and each channel, we estimated the power spectral density from 4 to 45 Hz (in steps of 1 Hz) in the same data used in the phase synchronization analysis. We analysed both absolute gamma power (30-45 Hz) and relative gamma power by dividing the sum of gamma power between 30 and 45 Hz by total power (sum of power from 4 to 45 Hz). We compared gamma power between eye-contact and control conditions using a paired t-test in each channel (using all participants included in the ciPLV analysis). Since no significant difference was found in any channel for both absolute (Supplementary Figure 3a) and relative (Supplementary Figure 3b) gamma power (all t-values < 2 and p >.05), there was no need to conduct a non-parametric cluster permutation (as we had no cluster observed in the real data). This analysis confirms that the effects we observed in the ciPLV are unlikely to derive from differences in gamma power between conditions.

Supplementary Figure 3: Differences in gamma power between eye-contact vs. control.

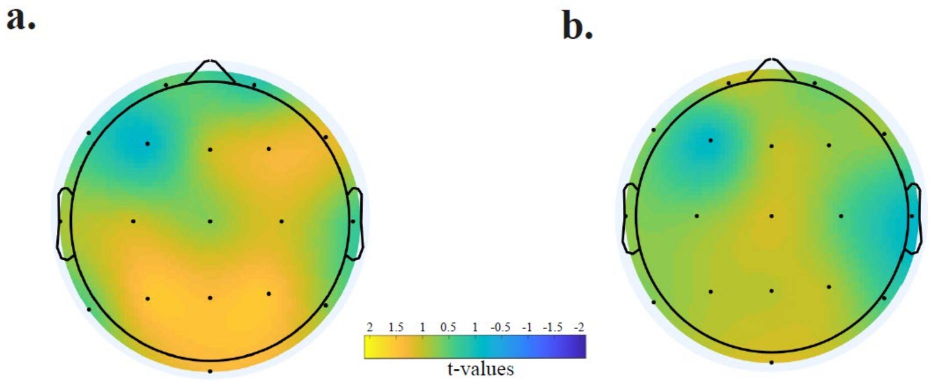

a. Topographical distribution of the differences in absolute gamma power between eye-contact and control expressed as t-values (paired t-tests); **b**. Same as in A but using relative gamma power as the dependent variable. All t-values were lower than 2 and corresponding p-values higher than .05.

Supplementary Note 4: Undirected Network Analysis

For the undirected connectivity data (ciPLV), we analysed the ROI based statistics for degrees and local efficiency of each ROI as well as the connectivity within each node. To do this, we averaged the degree (i.e. number of edges) and global efficiency of the network nodes (see Methods) between channels for each ROI (MP: mid posterior; MF: mid frontal, RTP: right temporo-parietal, LTP: left temporo-parietal, RF: right frontal, LF: left frontal). For network degree, we observed a main effect for ROIs ($F(7,686) = 37.409$, $p < .001$, partial $\eta^2 = .249$) and a main effect of friendship ($F(1,98) = 7.105$, $p = .009$, partial $\eta^2 = .068$), but no interaction between them ($F(7,686) =.521$, $p = .819$, partial $\eta^2 = .005$). Pairwise comparisons between ROIs showed that the midline areas (frontal and posterior) and the right parietal region showed higher degree than the other regions ($p < .001$). Midline frontal (MF) and posterior (MP) presented a higher degree compared to other regions ($p < .001$), but they did not differ from each other ($p < .121$). On Supplementary Figure 4a, the detailed contrasts are represented in the figure. Friends showed higher degree in all the ROIs, but that between the ROIs, the midline and right parietal regions were the most connected.

We did the same analysis for the local efficiency, calculated in the local efficiency of each node and averaged across ROI electrodes (Supplementary Figure 4b). Similar to what we found in relation to the degrees, we observed a main effect for ROIs ($F(7,686) = 48.150$, $p < .001$, partial $\eta^2 = .329$) and a main effect of friendship ($F(1,98) = 4.804$, $p = .031$, partial $\eta^2 = .047$), but no interaction between them ($F(7,686) =0.220$, $p = .954$, partial $\eta^2 = .002$). This suggests that the topography of the networks during eye-contact of friends and strangers was similar, but that friends showed a substantially more connected, hence efficient, network.

Regarding the rich-club structure of the network, we looked at how often each electrode behave as a rich-club node (as a proportion of all pairs, see Methods). The results (Supplementary Figure 3c) showed that the electrodes from midposterior, parietal, and central are the most likely to present rich club characteristics. In relation to each ROIs, the proportion of rich club electrodes was highest in the midposterior (81%), followed by right parietal (79%), left parietal (76%), midfrontal (72%), right centro-temporal (58%), left centro-temporal (55%) with the lowest values on right and left frontal areas (39% and 27% respectively).

Supplementary Figure 4. Network characteristics during eye-contact

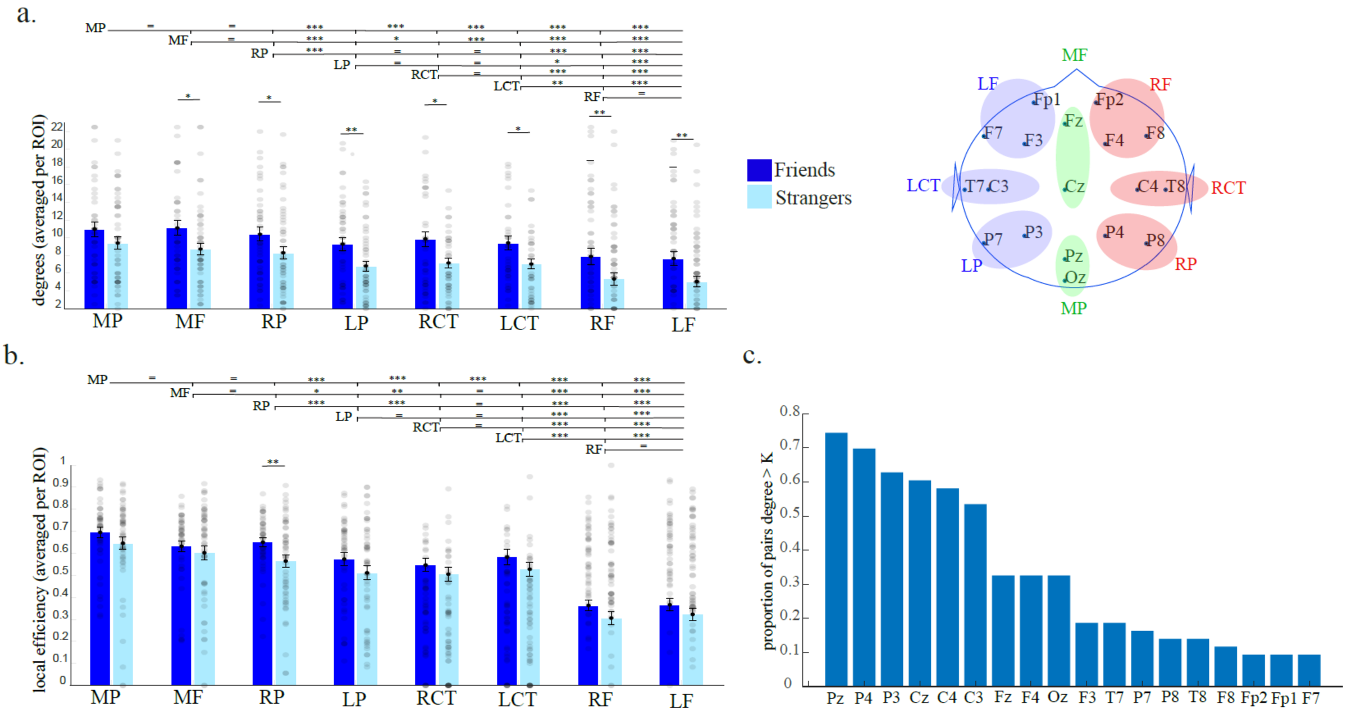

a. Degree of all connections (inter and intra-brain) of friends (dark blue) and strangers (light blue). The contrasts show the differences in the average degree between ROIs (top contrast lines) and between friends and strangers (lower contrast lines). Shaded dots represent the data for each participant (n = 100); **b.** Same as in **a** but with local efficiency instead. All contrasts have been Bonferroni corrected. Regions are: Midposterior (MP), Midfrontal (MF), right parietal (RP), left parietal (LP), right centro-temporal (RCT), left centro-temporal (LCT), right frontal (RF) and left frontal (LF). Error bars represent ±1 SEM. *** $p < .001$/** $p < 0.01$/ * $p < .05$. **c.** From all participant pairs, we calculated the proportion who showed a connectivity higher than K (see Methods) for each channel. Channel Pz was the most frequent rich club, followed by parietal and midcentral electrodes.

Supplementary Note 5: Directed Network Analysis

We analysed the network characteristics of our directed synchronization matrices (PSI). In order to evaluate whether eye-contact was associated with higher changes in inter- vs. intra-brain synchronization, we compared the network's average strength and density between them using a 2 (inter- vs. intra-brain) x 2 (leadership strength: strong vs. weak) mixed-design ANOVA on the absolute average z-scores. For the network strength, we observed that inter-brain connections increased significantly more than intra-brain connections during eye-contact ($F(1,96) = 12.252$, $p < .001$, partial $\eta^2 = .113$), but no interaction with nor main effect of leadership strength ($p > .2$). We observed the same effects for density, with a significantly higher proportion of inter-brain connections compared to intra- during eye-contact ($F(1,96) = 23.276$, $p < .001$, partial $\eta^2 = .195$), but no main effect of leadership strength nor interaction ($p > .1$). This demonstrates that eye-contact was associated with higher increase in inter-brain connectivity compared to intra-brain during eye-contact, independently of the leadership strength (Supplementary Figure 5).

Supplementary Figure 5: Intra- and inter-brain changes in directed connectivity (PSI) during eye-contact.

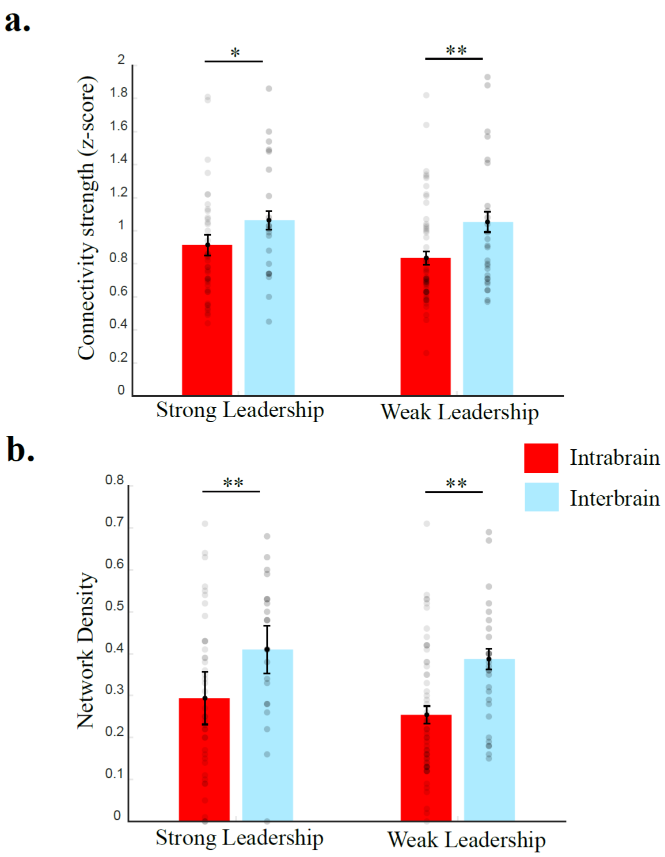

a. Absolute strength of the intra- (red) and inter-brain (light blue) connections during eye-contact. The strength is the absolute z-score against the control task. **b.** Network density (proportion of edges) for intra-brain (red) and inter-brain (light blue) connections (unsigned)

in pairs with strong vs. weak leadership. Shaded dots represent the individual data points in each condition ($n = 98$). Error bars represent ±1 SEM. *** $p < .001$/** $p < 0.01$/ * $p < .05$.

Supplementary Note 6: Local to Global efficiency analysis

The figure below shows the global efficiency in different ROIs which is used as a proxy to how much access each node has to the entire network.

Supplementary Figure 6: Global efficiency per ROI.

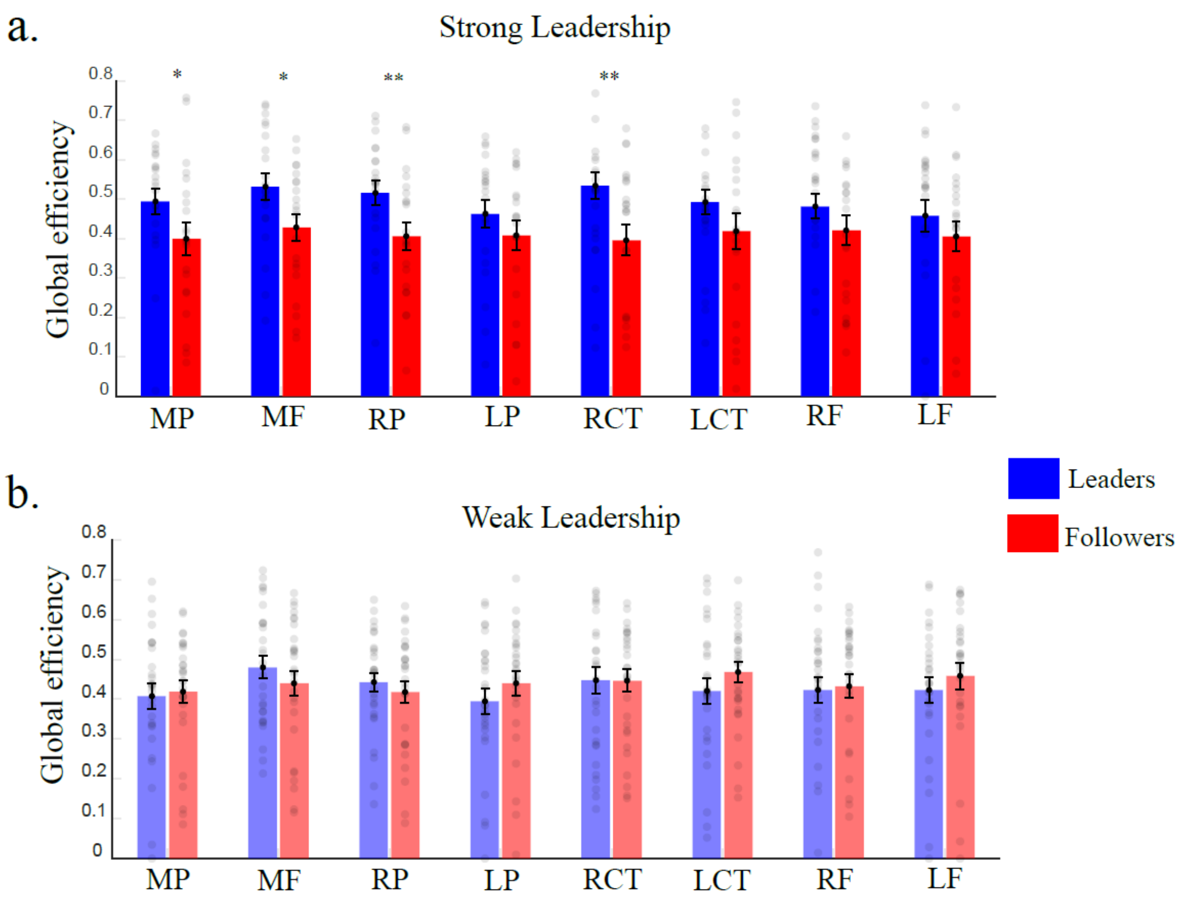

a. Global efficiency averaged over ROIs for leaders (blue) and followers (red) per ROI for pairs with strong leadership (darker). **b.** Global efficiency for leaders (blue) and followers (red) per ROI for pairs with weak leadership (lighter). Shaded dots represent the individual data points in each condition (n = 98). Error bars represent ±1 SEM. *** p < .001/** p < 0.01/ * p < .05.

Supplementary References

1. Latora, V. & Marchiori, M. Efficient behavior of small-world networks. *Physical review letters* **87**, 198701 (2001).
2. Fortunato, S. Community detection in graphs. *Physics reports* **486**, 75–174 (2010).
3. Newman, M. E. & Girvan, M. Finding and evaluating community structure in networks. *Physical review E* **69**, 026113 (2004).
4. Newman, M. E. Mixing patterns in networks. *Physical review E* **67**, 026126 (2003).
5. Zhou, S. & Mondragón, R. J. The rich-club phenomenon in the Internet topology. *IEEE Communications Letters* **8**, 180–182 (2004).
6. Colizza, V., Flammini, A., Serrano, M. A. & Vespignani, A. Detecting rich-club ordering in complex networks. *Nature physics* **2**, 110–115 (2006).
7. Newman, M. E. Networks. *Oxford university press* (2018)
