# Supplementary material for: Social synchronization of brain activity increases during eye-contact
Source: Commun Biol. 2022 May 4;5:412. doi: 10.1038/s42003-022-03352-6 (PMC9068716; doi:10.1038/s42003-022-03352-6)
Supplement: Supplementary file 3 — Reporting Summary [file 42003_2022_3352_MOESM3_ESM.pdf]

nature portfolio

Corresponding author(s): Caroline Di Bernardi Luft

Last updated by author(s): 20/02/2022

Reporting Summary

Nature Portfolio wishes to improve the reproducibility of the work that we publish. This form provides structure for consistency and transparency in reporting. For further information on Nature Portfolio policies, see our Editorial Policies and the Editorial Policy Checklist.

Statistics

For all statistical analyses, confirm that the following items are present in the figure legend, table legend, main text, or Methods section.

n/a	Confirmed	
☐	☒	The exact sample size (n) for each experimental group/condition, given as a discrete number and unit of measurement
☐	☒	A statement on whether measurements were taken from distinct samples or whether the same sample was measured repeatedly
☐	☒	The statistical test(s) used AND whether they are one- or two-sided<br>*Only common tests should be described solely by name; describe more complex techniques in the Methods section.*
☐	☒	A description of all covariates tested
☐	☒	A description of any assumptions or corrections, such as tests of normality and adjustment for multiple comparisons
☐	☒	A full description of the statistical parameters including central tendency (e.g. means) or other basic estimates (e.g. regression coefficient) AND variation (e.g. standard deviation) or associated estimates of uncertainty (e.g. confidence intervals)
☐	☒	For null hypothesis testing, the test statistic (e.g. F, t, r) with confidence intervals, effect sizes, degrees of freedom and P value noted<br>*Give P values as exact values whenever suitable.*
☒	☐	For Bayesian analysis, information on the choice of priors and Markov chain Monte Carlo settings
☒	☐	For hierarchical and complex designs, identification of the appropriate level for tests and full reporting of outcomes
☐	☒	Estimates of effect sizes (e.g. Cohen's d, Pearson's r), indicating how they were calculated

Our web collection on statistics for biologists contains articles on many of the points above.

Software and code

Policy information about availability of computer code

Data collection	The data was collected using the EEG devices Starstim 20 and the eye-trackers Eye-Tribe. The tasks were run in MATLAB and integrated using the MATNIC (Neuroelectrics) toolbox. The code used for the paradigm is available upon request to the corresponding author.
Data analysis	The EEG data was preprocessed using EEGLAB toolbox (Delorme & Makeig, 2004) in MATLAB (version R2018b). The non-parametric cluster permutation was performed in Matlab using custom written code. The network measures were calculated using Python (version 3.9.5 with Jupyter Notebook 6.4.0. The code and data are available in OSF (https://osf.io/y9cr2/?view_only=f9d6e2f7549f47dd8076787f499d4252).

For manuscripts utilizing custom algorithms or software that are central to the research but not yet described in published literature, software must be made available to editors and reviewers. We strongly encourage code deposition in a community repository (e.g. GitHub). See the Nature Portfolio guidelines for submitting code & software for further information.

Data

Policy information about availability of data

All manuscripts must include a data availability statement. This statement should provide the following information, where applicable:

- Accession codes, unique identifiers, or web links for publicly available datasets
- A description of any restrictions on data availability
- For clinical datasets or third party data, please ensure that the statement adheres to our policy

The main data (ciPLV and PSI) matrices and the data used for producing Figures 4a, 4d, 7a and 7b are available in OSF (https://osf.io/y9cr2/?view_only=f9d6e2f7549f47dd8076787f499d4252). Raw EEG is available from the corresponding author upon reasonable request.

Field-specific reporting

Please select the one below that is the best fit for your research. If you are not sure, read the appropriate sections before making your selection.

☐ Life sciences ☒ Behavioural & social sciences ☐ Ecological, evolutionary & environmental sciences

For a reference copy of the document with all sections, see nature.com/documents/nr-reporting-summary-flat.pdf

Behavioural & social sciences study design

All studies must disclose on these points even when the disclosure is negative.

Study description	This is a quantitative experimental study.
Research sample	Adult participants.
Sampling strategy	Random.
Data collection	The data was collected in laboratory settings, with 2 researchers and two study participants. Each participant had their EEG cap placed and prepared by one researcher. During the experiment, they sat in front of each other on opposite sides of a table. We monitored their eye-movements using two eye-trackers (positioned on the table, right in front of them). Each eye-tracker and EEG were connected to each PC (which were connected to each other).
Timing	The data collection started in September 2019 and ended in 10 of March 2020.
Data exclusions	One pair was excluded from the EEG analysis due to technical issues in the EEG recording, while five more pairs were excluded due to insufficient number of valid trials (<5 trials per condition) ascribed to motor artefacts (N = 100, 50 pairs). In the PSI analysis, another pair was excluded due to no leadership relationship (N = 98/ 49 pairs).
Non-participation	No participant declined participation or withdrew during the study.
Randomization	The sample were volunteers who contacted the researcher (via social media adverts and word of mouth). The allocation was random.

Reporting for specific materials, systems and methods

We require information from authors about some types of materials, experimental systems and methods used in many studies. Here, indicate whether each material, system or method listed is relevant to your study. If you are not sure if a list item applies to your research, read the appropriate section before selecting a response.

Materials & experimental systems

n/a	Involved in the study	
☒	☐	Antibodies
☒	☐	Eukaryotic cell lines
☒	☐	Palaeontology and archaeology
☒	☐	Animals and other organisms
☐	☒	Human research participants
☒	☐	Clinical data
☒	☐	Dual use research of concern

Methods

n/a	Involved in the study	
☒	☐	ChIP-seq
☒	☐	Flow cytometry
☒	☐	MRI-based neuroimaging

Human research participants

Policy information about studies involving human research participants

Population characteristics	Fifty-six pairs of adult participants (112 participants) took part in this experiment, all neurologically healthy adults. Of these, 27 pairs were friends (37 female, 17 male) with a mean age of 20.52 (SD = 1.59) years, while 29 were strangers (45 female, 13 male) with a mean age of 20.30 (SD = 2.30) years.
Recruitment	Via adverts on social media, campus and word of mouth.
Ethics oversight	Queen Mary University of London

Note that full information on the approval of the study protocol must also be provided in the manuscript.
